# Infection, Sepsis and the Inflammatory Response: Mechanisms and Therapy

**DOI:** 10.3389/fmed.2020.588863

**Published:** 2020-12-02

**Authors:** Dagan O. Lonsdale, Reya V. Shah, Jeffrey Lipman

**Affiliations:** ^1^Department of Clinical Pharmacology, St George's University of London, London, United Kingdom; ^2^Department of Critical Care, St George's University Hospitals National Health Service (NHS) Foundation Trust, London, United Kingdom; ^3^Department of Intensive Care, Royal Brisbane and Women's Hospital, Brisbane, QLD, Australia; ^4^University of Queensland Centre for Clinical Research, The University of Queensland, Brisbane, QLD, Australia; ^5^Division of Anaesthesiology Critical Care Emergency and Pain Medicine, NÎmes University Hospital, University of Montpellier, NÎmes, France

**Keywords:** infection, intensive & critical care, antibiotics, sepsis, Infectious disease

## Abstract

Sepsis secondary to bacterial infection remains a significant cause of morbidity and mortality globally. Recent decades have seen the evolution of international collaborations to improve care for these patients and identify areas for research. In this article we discuss the pathophysiology underlying the condition, review the current recommended management strategies, discuss areas of controversy, and highlight the need for ongoing research, particularly in diagnostics.

## Introduction

The global burden of bacterial infection is significant. In the UK, beta-lactams and macrolide antibiotics account for 25 million prescriptions annually, and global antibiotic prescriptions are in the billions (1, 2). Associated healthcare costs are considerable, accounting for over $30 billion (~8%) of US healthcare spending (3). At the severe end of the spectrum, sepsis is a common reason for admission to intensive care, accounting for as much as 30% of admissions to adult (4–6) and 12% of admissions to pediatric units (7, 8). Up to 70% of intensive care patients will receive at least one course of antibiotics during their stay, regardless of age (9, 10). Mortality from severe infection in adults and children remains as high as 25–30% (8, 11). In neonates, infection remains one of the most common causes of death worldwide (12–14). Understanding and managing infection is therefore a core skill for physicians and healthcare professionals. In this article, we will explore the pathology, diagnosis, and management of infection and sepsis, discuss some of the complexities and challenges.

## Infection, Sepsis, and the Inflammatory Response

The human response to an infection follows recognition of infectious pathogens by immune cells via pattern recognition receptors. The resulting cellular activation leads to the production of pro- (particularly IL-1 and TNFα) and anti-inflammatory mediators and the subsequent recruitment and activation of other immune cells (e.g., polymorphonucleocytes and B-cells) (15). In the normal host response to infection, equilibrium is reached between pro- and anti-inflammatory processes, bactericidal activity is maximized, alongside necessary phagocytosis, and repair of damaged tissue. In sepsis, this equilibrium is lost. Localized tissue response to infection becomes systemic and the inflammatory process becomes deleterious in its own right. Sepsis being formally defined as “life-threatening organ dysfunction caused by a dysregulated host response to infection” (16). Effects are seen across organ and tissue types. Endothelial damage results in loss of normal homeostasis, leading to fluid leak, and tissue oedema. In the vasculature there is vasodilatation mediated through several mechanisms such as the effect of acidosis on vascular smooth muscle, induction of nitric oxide by inflammatory mediators, and adrenal insufficiency. The consequent drop in systemic vascular resistance may be compensated by increased cardiac output or compounded by myocardial depression–leading to hypotension and reduced tissue perfusion. In the lungs, fluid leak may impair gas exchange and progress to acute respiratory distress syndrome. Acute kidney injury may result from direct cytokine effects as well as damage to the microvasculature or reduced tissue perfusion and hepatic effects may further impair a dysregulated coagulation system (15, 17). In the brain, inflammatory mediators, hypoxia, and hypotension may all contribute to the evolution of encephalopathy. Blood dyscrasias are also common, with mechanisms not fully understood. It may be that the expansion in haematopoietic stem cells secondary to demand and the inflammatory state may actually lead to the production of dysfunctional stem cells, paradoxically leading to production of less mature cells like neutrophils (and subsequent neutropenia) (18).

The dysregulated immune response to infection is unpredictable, leading to a wide spectrum of clinical presentations, and time-course for the disease. Multiple attempts have been made to describe the clinical effects in a simplified manner that allows early recognition of the septic patient. Perhaps most notably with the concept of the systemic inflammatory response syndrome (19). The use of clinical signs (fever or hypothermia, tachycardia, tachypnoea) and readily available investigations (high or low white cell count) to identify patients with this complex physiology in as simple a manner as possible is attractive. However, the lack of specificity of the SIRS criteria to identify sepsis specifically and the prevalence of cases of sepsis that do not meet these criteria make it an imperfect diagnostic tool (20–22). This lack of specificity may well-contribute to the high prevalence of antibiotic prescriptions in hospitals and the ICU (9, 10).

## Diagnosing Sepsis

In an attempt to address some of the limitations of the SIRS criteria and accompanying definitions of sepsis, a new consensus definition for sepsis was proposed in 2016—life threatening organ dysfunction caused by infection (16). Organ dysfunction was defined as an increase in sequential organ failure assessment (SOFA) score of 2 or more points. Whilst this definition may increase the sensitivity of diagnosing sepsis and identify those with the greatest mortality risk, it remains non-specific and has not been universally adopted (23, 24). Sepsis remains a challenge to diagnose. The search for a specific biomarker in sepsis is yet to yield success, despite hundreds of potential molecules being identified (25–27). Blood cultures are often cited as a gold standard method of identifying pathogens, but the test remains flawed in its utility in managing the early stages of sepsis given the long turn-around time and frequency of contaminated and false-positive/negative samples (28). Clinicians must therefore use signs, symptoms and supportive investigations to identify or suspect infection. This may be straightforward in the coughing, hypoxic patient with lobar consolidation identified on a chest radiograph or challenging in an elderly patient for whom dementia contributes to non-specific symptoms. Supportive signs and investigations like temperature, white cell count and inflammatory biomarkers like C-reactive protein or procalcitonin may be raised in any stress response and early antibiotics, whilst important for treatment, may impair the identification of a pathogen from samples cultured after administration.

## Therapies

The decision on where to treat a person who has sepsis will largely depend on local hospital resources and the mode and route of presentation. Many cases will present to the emergency department directly but management of cases identified in the community or in hospital inpatients merit consideration. Regardless of the route to identification of a case, consensus remains that early treatment of the infection and provision of supportive care improves outcome (24, 29, 30).

### Antimicrobials

The most recent surviving sepsis guidelines recommend administration of intravenous antimicrobials within 1 h of identification of sepsis (29). This recommendation is supported by multiple observational studies that show an association between delay in antimicrobial administration and increase in mortality (30–32). Choice of antimicrobial should be directed to provide coverage for the most likely pathogen that has caused the septic state. Mirroring the heterogeneity of the clinical presentation of sepsis, the range of possible pathogens is myriad and will be dependent on the patient, their presentation and previous medical history alongside local factors such as antimicrobial resistance patterns. Given this heterogeneity and the fact that pathogens are not usually identified at the time of diagnosis of sepsis, it is not possible to recommend a specific antibiotic or antibiotic combinations. Broad-spectrum coverage is usually therefore necessary, with choice of drug(s) directed at potential site of infection and coverage of risk factors. This may require the use of multiple agents, for example the combination of broad spectrum ß-lactam with vancomycin where MRSA is suspected and addition of antifungals where invasive fungal disease may be present (for example where immunocompromised state exists). Choice of agent should be taken in line with local prescribing guidelines. Dose and mode of antibiotic administration continues to evolve in the management of sepsis. The one-size fits all dosing strategy used in the management of community infection cannot hold in the management of sepsis where profound alterations to antimicrobial pharmacokinetics are likely secondary to both organ dysfunction and changes in volume of distribution from altered volume status (secondary to the disease and fluid resuscitation in its management) (29, 33). Whilst organ dysfunction associated with sepsis can lead to accumulation of antimicrobials, this is not universal. Altered antimicrobial pharmacokinetics in sepsis has been shown to reduce plasma concentration to below levels considered therapeutic (34, 35) and failure to achieve therapeutic plasma concentrations has been associated with failure of therapy (36). It is therefore recommended that dose and frequency of administration are considered and individualized from the outset of treatment. These considerations should include the pharmacokinetic-pharmacodynamic relationship between antimicrobial and pathogen (Table 1) such that dose and dosing regimen are optimized. For example, the pharmacokinetic-pharmacodynamic index associated with successful bacterial kill of ß-lactams is time with drug concentration above the minimum inhibitory concentration of the target bacteria. Frequent (and adequate) dosing is therefore required to maintain an appropriate concentration time profile for ß-lactams. A phase III clinical trial is ongoing into the utility of continuous infusions (39). Completely individualized treatment courses are difficult to achieve without rapid therapeutic drug monitoring, which is often not routinely available, save perhaps for the monitoring for harm of aminoglycosides. Duration of therapy should minimize exposure to broad-spectrum antibiotics where possible. This should include switching to narrow spectrum agents where pathogens and susceptibility are identified and minimizing the duration of multi-agent regimens by, for example, reducing to single agent cover as shock resolves and there is clinical improvement. Stewardship of antimicrobials in this way will minimize the potential for emergence of resistant pathogens. Indeed, such stewardship should be considered *as* important a place in therapy as initial choice and dose of antibiotics, since infection with multi-drug resistant pathogens is associated with increased hospital length of stay and mortality (40, 41) and alteration in commensal microbiome to resistant pathogens is rapid—as quickly as within 1 day of exposure to imipenem in one study (42).

**Table 1:** Commonly used antibiotics and the pharmacokinetic-pharmacodynamic index associated with therapeutic success.

**Antibiotic class**	**Pharmacokinetic-pharmacodynamic index**
Aminoglycosides	C_MAX_:MIC
β-lactams	T>MIC
Fluroquinolones	C_MAX_:MIC
Glycopeptides	AUC/MIC
Macrolides	AUC/MIC

*Table adapted from Roberts and Lipman (37) and Asin-Prieto et al. (38). C_MAX_, maximum concentration; MIC, minimum inhibitory concentration of suspected pathogen; T, time; AUC, area under the time-concentration curve*.

### Source Control

Where sepsis is caused by a focus of infection that is amenable to source control through surgical or other intervention (e.g., removal of infected indwelling catheters), source control should occur as quickly as practical (within 6–12 h) (43–46).

### Fluid Therapy

Whilst there are few clinicians who would advocate removing fluids from the armory of sepsis management, the choice, amount and rate of administration of fluid remains controversial and it is an area of need for ongoing research. The principle of restoring a circulating volume to provide adequate tissue perfusion in the vasodilated and fluid shifting septic state is sound, but for some patients with sepsis cardiac output may be elevated and tissue blood flow may be increased and oxygen delivery satisfactory. The one size fits all fluid dose of 30 ml/kg recommended in the most recent surviving sepsis guidelines is, therefore, controversial. Indeed, by the guide's own acknowledgment the evidence to support this recommendation is “low quality” (29) and there is evidence of harm of over resuscitation in some settings (5, 47). A gold standard guide to adequacy of resuscitative volume does not exist. Raised serum lactate, whilst associated with worse outcomes in sepsis, may be elevated in the setting of catecholamines (endogenous or exogenous), hepatic failure, or altered cellular metabolism in sepsis (29, 48). It is therefore an imperfect guide to volume state. Indeed, a recent trial found no difference in mortality outcome between septic patients in whom fluid resuscitation targeted lactate normalization vs. normal capillary refill time (49). It would seem prudent, as with recommendations on antimicrobial and vasopressor administration, that fluid dosing in sepsis should be personalized. This entails the use of small bolus fluid administration (250–500 mL) with frequent reassessment. The perfect assessment modality does not exist, simple measures like capillary refill time or straight leg raise tests may well be as useful as more complex or invasive assessment modalities such as point of care ultrasound or haemodynamic monitoring software.

Crystalloids infusions are favored over albumin and synthetic colloids as the initial fluid of choice, although there is insufficient evidence to guide choice of crystalloid in sepsis. Balanced solutions are often preferred to high chloride (0.9% sodium chloride) infusions but direct head to head trials in sepsis are missing. Albumin may be considered as a fluid choice, but high-quality evidence of benefit over crystalloids is lacking (29, 50, 51).

### Target Blood Pressure

A target mean arterial pressure has of 65 mmHg has been advocated in sepsis (29). This target is based on the principal of reduced tissue perfusion with lower pressures. Trials of higher target mean arterial pressure have not shown clinically meaningful benefit and some evidence of harm from adverse drug effects (52–54). However, it may be that a lower target is acceptable. In the recently published “65-trial,” a lower target mean arterial pressure of 60 mmHg was tested against the 65 mmHg clinical standard in older (over-65) critically ill patients with vasodilatory shock (55). There was no mortality difference shown between the two targets. The results of this study has been cited as supporting personalisation of pressure targets, accepting lower bound of 60 mmHg in some instances (56), although this has not been adopted in the recent COVID-19 interim guidance issued by the surviving sepsis group (57).

### Vasopressors

Norepinephrine is generally accepted as the first-line vasoactive agent in fluid-refractory septic shock in adults (29), with the therapeutic aim of restoring mean arterial pressure and tissue perfusion. Superiority of norepinephrine over dopamine has been demonstrated in systematic reviews, both in terms of survival outcome and reduced adverse effects (58, 59). The surviving sepsis continue to include a recommendation for the use of dopamine as an alternative in select patients with low risk of tachyarrhythmias, although the guidelines do not make recommendations on how to select these patients (29). Vasopressin may be considered as a second line agent in vasodilatory shock that persists despite norepinephrine. There is no mortality benefit of one drug above the other and vasopressin is probably best considered norepinephrine sparing (29, 60, 61). Data guiding the norepinephrine dose at which a second agent should be instituted is lacking. The surviving sepsis authors advocate an upper dose limit of 0.03 units/min of vasopressin, this was the dose used in the intervention arm of the VASST trial (61). The more recent VANISH trial used an upper limit of 0.06 units/min but showed numerically higher incidence of digital and mesenteric ischaemia compared with norepinephrine doses (60). Epinephrine is an alternative second line agent. For the subset of patients with myocardial impairment and low cardiac output secondary to sepsis, the surviving sepsis guidelines suggest considering dobutamine as an inotrope.

### Other Potential Therapies

A plethora of additional agents have been advocated as tools in the armory for the treatment of sepsis and shock associated with sepsis. Corticosteroids, immunoglobulins, vitamin C (with or without thiamine) are some more common recent examples. None have been shown to provide meaningful mortality benefit in high quality randomized controlled trials. Corticosteroids retain a place for those with adrenal insufficiency (chronic steroid use, Addison's disease etc.). Some also advocate corticosteroid use (200 mg/day for 7-days) in septic shock refractory to fluid and vasopressor therapy, citing shorter time to resolution of shock and shorter ICU stay (62, 63). There does not appear to be a risk of increased secondary infection with this approach, although hyperglycaemia and hypernatraemia are more common with steroid use (63). Multiple trials investigating the effect of vitamin C in sepsis are ongoing.

### Supportive ICU Care

Septic patients should receive standard ICU care bundles that include venous thromboembolism and stress ulcer prophylaxis, glucose control and sedation care for ventilated patients.

## Conclusion and Future Work

Sepsis remains a challenging condition to manage. Heterogeneity of presentation, similarity to other inflammatory states and disease time-course makes diagnosis challenging and mortality remains high despite global efforts in improving treatment. International collaboration has been extraordinary and provides a good starting point for management. Treatment recommendations are not without controversy and are probably best used as a platform to design personalized treatment regimens, rather than rigidly sticking to protocols. Ongoing work must focus on improved diagnostics, alongside novel therapeutics. The need for an sensitive and specific biomarker seems most pressing, as it would enable appropriate management for the individual patient, assist in antimicrobial stewardship to protect drugs for the population and provide the ability to more accurately recruit participants with true sepsis to interventional trials.

## Author Contributions

All authors listed have made a substantial, direct and intellectual contribution to the work, and approved it for publication.

## Conflict of Interest

JL received honoraria for lectures from MSD and Pfizer, and Institutional support from MSD. The remaining authors declare that the research was conducted in the absence of any commercial or financial relationships that could be construed as a potential conflict of interest.
